# Revelation of Osseous Syphilis Following Gun Shot Wounds

**Published:** 1916-03

**Authors:** 


**Revelation of Osseous Syphilis Following Gun Shot Wounds.** H. Toussaint, Soc. de Chir., 1915, page 477 notes tardy healing due to syphilitic lesions pre-existing or determined by the local injury.

